# Supplementary material for: Detraque: Dynamic execution tracing techniques for automatic fault localization of hardware design code
Source: PLoS One. 2022 Sep 16;17(9):e0274515. doi: 10.1371/journal.pone.0274515 (PMC9481022; doi:10.1371/journal.pone.0274515)
Supplement: Attachment — Submitted filename: Response to Reviewers.pdf [file pone.0274515.s001.pdf]

# Response to the comments given by the editors and reviewers of Manuscript: PONE-D-22-09808, Minor revision

## (Detraque: Dynamic Execution Tracing Techniques for Automatic Fault Localization of Hardware Design Code)

Dear Editors and Reviewers,

We would like to thank you and the anonymous reviewers for their constructive comments and recommendations to improve the manuscript. We have addressed all the comments. The details of our response are below. We are happy to answer any further questions that might arise.

Yours Sincerely,

Jiang Wu, Zhuo Zhang, Jianjun Xu, Jiayu He, Xiaoguang Mao, Xiankai Meng, and Panpan Li

---

## Response to Journal Requirements:

***Comment:*** *Upon re-submitting your revised manuscript, please upload your study's minimal underlying data set as either Supporting Information files or to a stable, public repository and include the relevant URLs, DOIs, or accession numbers within your revised cover letter.*

**Response:** We have upload our study's minimal underlying data set to https://github.com/wndif/Benchmark_Detraque.git. We also included the Github link on Page 3 of the article.

## 1. Response to Reviewer #1's comments:

***1.1 Comment:***
*Please review your English grammar. For instance, "we" have been used many times.*

**Response:** We very much appreciate your constructive recommendations. In summary, we have made the following major changes according to your advice in this revision: we first passed a manual grammar check and then used the tool "Grammarly" to further check the grammar. At the same time, we also replaced a large number of "we" with more appropriate pronouns.

***1.2 Comment:***

*All the figures are not available in the text, and they all are available in the end which is difficult to follow.*

**Response:** Sorry for making it difficult for you to read. Separation of paper text and figures is a submission requirement of PLOS ONE, so figures are attached at the end of the text when submitting. It was merged together in the final published version. Meanwhile, we have also adjusted the position of the figures in the text and further marked the reference to the figures in the text, so as to help readers better grasp the content of the figures.

***1.3 Comment:***

*Please check the format of your references. For instance, Reference 32 has capital letters which is different than others. You have used "//". Please check again?*

**Response:** We very much appreciate your constructive comments and sorry for our inappropriate illustration. We have revised your problem with "Reference 32" and "//" on Section Reference. We then carefully checked the format of all references and refined them.

***1.4 Comment:***

*Please check your table format. For example, Table 3 in page number 10.?*

**Response:** Thank you for the advice. We have revised the "Table 3 in page number 10". We then carefully checked the format of all tables and refined them.

## 2. Response to Reviewer #2's comments:

***2.1 Comment:***

*It is a very valuable contribution. This will be more beneficial if some related material from previous issues of the journal in the literature review will be added, however, the literature review has done rigorously.*

**Response:** We very much appreciate your constructive comments and sorry for our inappropriate illustration. According to your advise, we have added two articles from previous issues of the journal and noted them in the revised version, they are:

*1.Qu N, You W. Design and fault diagnosis of DCS sintering furnace's temperature control system for edge computing[J]. PloS one, 2021, 16(7): e0253246.*

*2. Xiao Y, Wang K, Liu W, et al. Research on rapier loom fault system based on cloud-side collaboration[J]. PloS one, 2021, 16(12): e0260888.*
